# *KEGGViewer*, a BioJS component to visualize KEGG Pathways

**DOI:** 10.12688/f1000research.3-43.v1

**Published:** 2014-02-13

**Authors:** Jose M. Villaveces, Rafael C. Jimenez, Bianca H. Habermann

**Affiliations:** 1 Max Planck Institute of Biochemistry, Am Klopferspitz 18, 82152, Germany; 2 European Bioinformatics Institute, Wellcome Trust Genome Campus, Hinxton, Cambridge, CB10 1SD, UK

## Abstract

**Summary:** Signaling pathways provide essential information on complex regulatory processes within the cell. They are moreover widely used to interpret and integrate data from large-scale studies, such as expression or functional screens. We present KEGGViewer a BioJS component to visualize KEGG pathways and to allow their visual integration with functional data.

**Availability: **KEGGViewer is an open-source tool freely available at the BioJS Registry. Instructions on how to use the tool are available at http://goo.gl/dVeWpg and the source code can be found at http://github.com/biojs/biojs and DOI: 10.5281/zenodo.7708.

## Introduction

Networks and network-based techniques are widely used in systems biology to model biological processes such as gene regulation, protein interactions and signaling pathways. Signaling pathways in particular, provide an understanding of cell dynamics by describing step by step the temporal interactions that a group of molecules or metabolites undergo in order to control one or more cellular functions.

Different attempts have been made to store and aid the retrieval and analysis of signaling pathways. For example the *Kyoto Encyclopedia of Genes and Genomes (KEGG)* ^1^ contains a large collection of manually curated pathway maps. *Panther Pathway* ^2^, as another example, provides access to a number of mainly signaling pathways, subfamilies and protein sequences mapped to individual pathway components.

KEGG is widely used by researchers to retrieve pathway information. Pathway maps in KEGG can be downloaded as static PNG images or alternatively as KEGG Markup Language (KGML) files (free of charge for academic use). KGML is an XML-like format that describes a pathway, its components and relationships and can, for instance, be used to visualize pathways ^3^, generate systems biology models ^4^ or perform network analysis ^5^.

Large-scale techniques like expression arrays, deep sequencing or proteomics allow monitoring the relative or absolute level of expression for a large number of genes simultaneously. However, expression profiling by itself is not sufficient to understand the exact role of a set of genes in a biological process. In order to gain new insights into the regulatory relationships of differentially regulated genes, expression profiles from a large-scale study can be integrated with signaling pathways.

Here, we present *KEGGViewer*, software that allows visual integration of KEGG pathways and expression profiles. We have coded *KEGGViewer* in BioJS ^6^, a JavaScript library that holds components for visualizing biological data on the web. The *KEGGViewer* component is open source and freely available at http://goo.gl/dVeWpg.

## The *KEGGViewer* component

**Figure 1.**
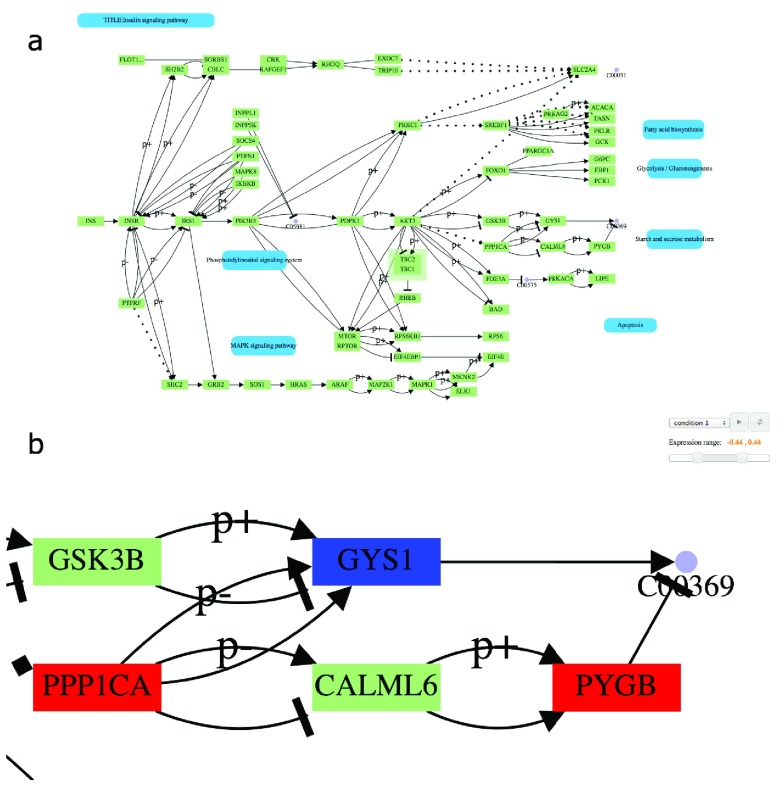
( **a**) *KEGGViewer* rendering of the insulin signaling pathway. Pathway components can be manually repositioned. Genes and pathways are represented as green and blue boxes respectively while purple dots represent chemical compounds. Relationships represent reactions e.g. activation, inhibtion or phosphorilation. ( **b**) Zoomed view of the insulin signaling pathway. *Condition 1* is selected in the control panel (top right) and the expression range is set to consider expression levels between -0.43 and 0.43 to be non differentially expressed. Genes *PPP1CA* and *PYGB* in red are upregulated while *GYS1* in blue is downregulated. *GSK3B* and *CALML6* in green are non differentially expressed genes. The purple dot *C00369* represents Starch.

To run *KEGGViewer* (i) a target DIV ID (unique identifier) to render the pathway, (ii) a KEGG pathway ID and (iii) a proxy URL to bypass the same domain policy constraint in JavaScript are required. The following code snippet illustrates how to initialize the component:



                    var instance = new Biojs.KEGGViewer ({
    target: “example”,
    pathId: “hsa04910”,
    proxy: “proxy.php”
});
                


With that input, *KEGGViewer* queries the KEGG API ^7^ in order to obtain the KGML-formatted KEGG pathway. Once retrieved, the KGML file is parsed by *KEGGViewer* and the pathway is rendered using *Cytoscape.js* ^8^ Figure 1a).

To contextualize regulatory relationships between a predefined set of genes, *KEGGViewer* can integrate userprovided gene expression data in a pathway ( Figure 1b). For this, the expression values must be handed over to *KEGGViewer*. The following code shows how to initialize the component to overlay expression data:



                    var instance = new Biojs.KEGGViewer({
    target: “example”,
    pathId: “hsa04910”,
    proxy: “proxy.php”,
    expression:{
        upColor:’red’,
        downColor:’blue’,
        genes: [’hsa:2998’, ’hsa:5834’,
        ’hsa:5499’, ’hsa:2194’],
        conditions: [
          {
              name:’condition 1’,
              values: [–1, 0.5, 0.7, –0.3]
          },
          {
              name:’condition 2’,
              values: [0.5, –0.1, –0.2, 1]
          },
          {
              name:’condition 3’,
              values: [0, 0.4, –0.2, 0.5]
          }
        ]
    }
});
                


The *expression* parameter defines the color to highlight up- and down-regulation, the genes affected and the different experimental conditions, in which expression values were obtained for the affected genes ( Figure 1b).

By providing expression data to *KEGGViewer*, the tool is able to (i) highlight genes according to their expression values in each experimental condition, (ii) allow users to change the threshold parameters for up- and down-regulation, and (iii) visualize expression changes under different experimental conditions as a slideshow.

More details on how to use *KEGGViewer* can be obtained from the BioJS Registry in http://goo.gl/dVeWpg.

## Conclusions

*KEGGViewer* is a simple, web-based component for visualization of KEGG pathways and integration of user-provided expression data on pathway information. It follows the principles of reutilization, sharing and development behind BioJS. *KEGGViewer* is easy to integrate in any website and provides functionality to interact with other JavaScript components. As a BioJS component, *KEGGViewer* is easy to extend allowing changes to be made or new functionality to be included.

## Software availability

Zenodo: KEGGViewer, a BioJS component to visualize KEGG pathways, doi: 10.5281/zenodo.7708 ^9^

GitHub: BioJS, http://github.com/biojs/biojs
