# Supplementary material for: KEGGViewer, a BioJS component to visualize KEGG Pathways
Source: F1000Res. 2014 Feb 13;3:43. [Version 1] doi: 10.12688/f1000research.3-43.v1 (PMC3954160; doi:10.12688/f1000research.3-43.v1)
Supplement: Peer review history

# Peer review: Referee response for version 1

**Published:** 2014-02-28

**Authors:** Alexander Pico

**Affiliations:** 1 Gladstone Institutes, San Francisco, CA, USA

The BioJS library of components has a lot of potential. It's encouraging to see a diversity of interactive viewers already registered with BioJS. The intersection of modern JavaSript (JS) components with network biology in particular is ripe for development to bring powerful perspectives on massive biomedical datasets to researchers. I decided to critique this article introducing the BioJS *KEGGViewer *from three points of view to acknowledge the broad set of use cases *and challenges* this work takes on. While there are a number of things to improve upon (as always) and a few points requiring clarification, the project is a nice addition to the BioJS library and may provide a useful data visualization option when deployed with a complementary set of web tools for selecting pathways, managing datasets and viewing details.

**Generic User:**

- The "play" feature is great for comparing conditions. Nicely done!
- Panning is tricky, I seem to have to hold cmd, click, pause, and then drag. Without the 'pause' I invoke a selection tool.
- There is no additional information or link-outs when you click on a node; only the gene symbol is provided.
- There is no interface for accessing the data values underlying the visualization. There is a disconnect between the web developer who sets up the viewer with all the underlying expression data and the end-user who views the data with only limited access and controls.

**Biomedical Researcher:**

- The default expression range appears to be set at min-min, which results in all data values visualized as up-regulation. I would recommend default values centered on 0 in addition to support for user-provided parameters.
- Unfortunately, the parameter names and value ranges for data overlays are unnecessarily restricted to "expression", "upColor" and "downColor". A generic solution for data overlay that could work with any type of data ( *KEGGViewer *shouldn't care if it's expression or not) and color gradients or discrete mapping options would be much more useful.
- All of these sorts of options are in fact already available in closely related tools (also free and open source, and which I happen to work on) that the authors neglected to cite: PathVisio [ 1] and Cytoscape [ 2]. These projects have both Java and JavaScript flavors. The JS version of Cytoscape was obviously used and cited in this work, but the Java version with its built-in data import, style and overlay options -- as well as KEGG import -- was missed. 
- Speaking of KEGG, I'm dubious about the blanket statement that it is "free of charge for academics". It's a complicated situation that I know many colleagues are unclear about, so I think it's important to describe it thoroughly. According to their own website [ 3], "Academic users who utilize KEGG for providing academic services are requested to obtain a KEGG FTP subscription for organizational use, which includes a proper license agreement." This leads to a licensing agent with various paid subscription options [ 4, 5]. The KEGG API, which *KEGGViewer* uses, is indeed freely provided for academic use, but only for individual downloads. Bulk downloads, such as those required to do analysis of over representation or enrichment, are explicitly forbidden and require a KEGG FTP subscription [ 6].

**Software Developer:**

- It is unfortunate that the EBI host site has resources in conflict with the *KEGGViewer*. This seems counter to the whole point of BioJS and should be addressed in future releases of the EBI web site, cytoscape.js and/or *KEGGViewer* (whichever CSS is the most intrusive or classes least specific).
- Beyond a bit of copy/paste JS (including a 5-level deep JS object), asking users to host a php proxy will likely turn some away. Is there any way around this? 

References **

1. http://pathvisio.org

2. http://cytoscape.org

3. http://www.kegg.jp/kegg/legal.html

4. http://www.bioinformatics.jp/en/keggftp.html

5. http://www.pathway.jp/licensing/commercial.html

6. http://www.kegg.jp/kegg/rest/

I have read this submission. I believe that I have an appropriate level of expertise to confirm that it is of an acceptable scientific standard.

# Peer review: Referee response for version 1

**Published:** 2014-02-25

**Authors:** Hedi Peterson, Priit Adler

**Affiliations:** 1 University Medical Center (CMU), University of Geneva, Geneva, Switzerland; 2 University of Tartu, Tartu, Estonia

*KEGGViewer* is a BioJS component for easy visualization of KEGG pathways. Although the article is quite short it provides all the essential information about the BioJS component for KEGG pathway visualization and points interested users to the source code for its implementation.  We do however have some minor comments about the text:

1. The emphasis of signaling pathways is not reasoned enough in the text. KEGG has signaling pathways, but it is so much more (e.g. metabolism, information processing, diseases, etc). For the usage of the given component it makes no difference between pathway classification, this should be clarified.
2. KEGG also has information about metabolites but this has not been mentioned in the text nor in the documentation of the component. I would assume that *KEGGViewer* is capable of handling metabolite data as well, but it would be nice to have it specified in the text and/or in the documentation of the component.
3. Although *KEGGViewer* is an easy plugin for visualizing KEGG pathways it is not a unique way for visualizing user data and alternative options could be mentioned in the Introduction section. KEGG itself allows for user data mapping, for example, KEGGanim is a special web tool for mapping metabolite and gene expression data to the pathways. Other alternatives that could be mentioned include Reactome, which allows expression analysis from user provided data.
4. Although the BioJS *KEGGViewer* component page has enough information to create working examples of the component, not all the requirements are self-explanatory (missing UI icons, display problems on certain mac chrome versions, expression range setup bar is confusing and it could be set to a default state at 0,0, the proxy setup is confusing and needs better documentation).
5. Currently, the description of parameters and options allows only basic usage. To make the component usable for a wider range of users and to display it's full power, the authors will have to considerably update the component description with additional details and 3-4 use cases. 

We have read this submission. We believe that we have an appropriate level of expertise to confirm that it is of an acceptable scientific standard.
